# Airway Remodeling in Asthma

**DOI:** 10.3389/fmed.2020.00191

**Published:** 2020-05-21

**Authors:** Kenneth P. Hough, Miranda L. Curtiss, Trevor J. Blain, Rui-Ming Liu, Jennifer Trevor, Jessy S. Deshane, Victor J. Thannickal

**Affiliations:** Division of Pulmonary Allergy and Critical Care Medicine, Department of Medicine, University of Alabama at Birmingham, Birmingham, AL, United States

**Keywords:** asthma, epithelium, airway remodeling, mesenchyme, immune cells

## Abstract

Asthma is an inflammatory disease of the airways that may result from exposure to allergens or other environmental irritants, resulting in bronchoconstriction, wheezing, and shortness of breath. The structural changes of the airways associated with asthma, broadly referred to as airway remodeling, is a pathological feature of chronic asthma that contributes to the clinical manifestations of the disease. Airway remodeling in asthma constitutes cellular and extracellular matrix changes in the large and small airways, epithelial cell apoptosis, airway smooth muscle cell proliferation, and fibroblast activation. These pathological changes in the airway are orchestrated by crosstalk of different cell types within the airway wall and submucosa. Environmental exposures to dust, chemicals, and cigarette smoke can initiate the cascade of pro-inflammatory responses that trigger airway remodeling through paracrine signaling and mechanostimulatory cues that drive airway remodeling. In this review, we explore three integrated and dynamic processes in airway remodeling: (1) initiation by epithelial cells; (2) amplification by immune cells; and (3) mesenchymal effector functions. Furthermore, we explore the role of inflammaging in the dysregulated and persistent inflammatory response that perpetuates airway remodeling in elderly asthmatics.

## Introduction

Asthma is a widely prevalent disease. In the United States, 13.4% of adults aged 18 years and older, and 11.6% of children are diagnosed with asthma. These rates are higher in adult females (15.2%) and male children (13%); in general, asthma prevalence is higher in minority populations and populations with low socioeconomic status^1^ (1). According to the most recent Global Initiative for Asthma (GINA) guidelines, asthma is defined as “a heterogeneous disease, usually characterized by chronic airway inflammation.” It is characterized by respiratory symptoms such as wheezing, shortness of breath, chest tightness, and cough that vary over time and in intensity, together with variable expiratory airflow limitation^2^. The variable airflow limitation in asthmatics is due to a combination of bronchoconstriction, airway edema, mucus secretion, airway hyper-responsiveness, and airway remodeling (2)^1^. However, variable airflow limitation may progress to persistent airflow limitation or fixed airway obstruction in a subset of patients (3).

## Epidemiology

Among the general population, asthma accounts for 30–50% of those individuals with fixed airway obstruction (4–6); in severe or difficult-to-treat adult asthmatics, 55–60% have fixed airway obstruction (7, 8). Airway remodeling may explain persistent airflow obstruction present in some asthmatic patients, attributed to goblet cell hyperplasia, decreased epithelial cell and cartilage integrity, subepithelial collagen deposition with increased thickness of the reticular basement membrane, increased airway smooth muscle mass and angiogenesis of the airways (9–15). This is present in asthmatics with mild disease (16), but tends to worsen in parallel with increasing disease severity (13, 17). Importantly, onset of airway remodeling has been identified in pre-school children as young as 1-year-old (18, 19) and in school-age children, persisting through adulthood. Thus, airway remodeling in some patients may occur early in the disease process (20–22). Conversely, adult asthma patients with minimal airway remodeling similar to healthy controls have also been identified (23, 24), and adult mild asthmatics acutely increase parameters of airway remodeling with exposure to asthma triggers (25). Thus, while airway remodeling may be a consequence of inflammation, the heterogeneity in its presentation suggests that it should not be assumed to occur downstream of a single (or central) mechanism. Ultimately, these varying mechanisms will illuminate our understanding of asthma endotypes.

## Asthma Endotypes

The field of personalized medicine in asthma care has benefitted greatly from the recognition that “asthma” refers to an umbrella term encompassing a range of clinical presentations (26). An aspirational goal of this phenotyping process is to eventually link clinical phenotype to molecular mechanisms, defining an “endotype” that would predict response to therapy (27).

One of the first approaches to phenotyping asthmatics was to evaluate sputum cellularity as an indirect readout of airway inflammation. Four subgroups of adult asthmatics were identified: eosinophilic asthma, neutrophilic asthma, mixed granulocytic asthma with both sputum eosinophils and neutrophils, and paucigranulocytic asthma with neither (28, 29). In multiple cohorts, the distribution of asthma favors eosinophilic (40–50%) and paucigranulocytic (30–50%) airway inflammation, with only 10–20% of patients manifesting neutrophilic asthma (28–32).

Unbiased identification of phenotypic clusters of asthmatics have incorporated objective clinical and morphometric parameters, including peripheral blood eosinophil counts, sputum cellularity, history of atopy, age of onset of asthmatic disease, body mass index, asthma control questionnaire, and presence of fixed vs. variable airflow obstruction (33, 34). These parameters when applied with unbiased clustering algorithms to large patient cohorts [SARP (Severe Asthma Research Program), ADEPT (Airways Disease Endotyping for Personalized Therapeutics), and U-BIOPRED (Unbiased BIOmarkers in PREDiction of respiratory disease outcomes)] consistently identify four to six clinically defined clusters (35–37). From these studies, a consensus has arisen that, at a minimum, asthma includes “Th2-high” and “Th2-low” disease subclusters. Th2-high includes: early-onset allergic asthma, late-onset steroid-resistant eosinophilic asthma, and aspirin-exacerbated respiratory disease (AERD). Th2-low tends to be steroid-resistant with either neutrophilic or paucigranulocytic inflammation and it is further classified by obesity, smoking, or onset after 50 years of age (38).

In contrast to unbiased clustering methods utilized above, the most widely used and easily implemented parameters in clinical use to assess asthma phenotype are the presence of sensitization to perennial aeroallergens, peripheral eosinophil count, fixed airway obstruction, and fractional exhaled nitric oxide (FENO) (39). This classification scheme differentiates Th2-high asthma, which is amenable to treatment with anti-IgE, anti-IL-5 or anti-IL-5Rα, and anti-IL-4Rα therapy (40, 41), from Th2-low asthma (42). In the context of cigarette use history and associated onset of asthma symptoms, patients with fixed airway obstruction may also be classified as “asthma-COPD (chronic obstructive pulmonary disease) overlap” (43).

### Airway Remodeling Endotypes

Airway remodeling may provide further specification of asthma endotypes. Post-mortem studies of asthmatic patients reveal that airway remodeling can affect both large and small airways (44, 45). However, invasive assessments of remodeling predominantly evaluated endobronchial biopsies of proximal airways rather than small airways (46), limiting our understanding of the small airway changes that are relevant to air trapping and may be relevant to airway remodeling (6). Non-invasive means of evaluating airway remodeling are needed that correlate with airway morphometric analyses. The gold standard of airway remodeling requires bronchial biopsy and direct assessment of lung tissue (6). Quantitative Computerized Tomography (CT) imaging of lung is a non-invasive means of assessing airway remodeling. In adult asthmatics, comparison of airway biopsies with CT morphometrics indicates a good correlation between airway wall volume and increased reticular basement membrane thickness (47). However, this association was not reproduced in a pediatric severe asthma cohort undergoing endobronchial biopsy (48) in spite of consistent identification of bronchial wall thickening on CT in cohorts of children with difficult-to-treat asthma (49). Regardless, CT imaging is now increasingly used to assess airway remodeling in adult asthmatics (50–52).

Asthma is often characterized as childhood-onset or adult-onset (53, 54). Childhood-onset asthma typically occurs prior to 12 years of age, whereas 40% of adult asthmatics report symptoms after 40 years of age (55, 56). Childhood-onset asthma is predominantly atopic and eosinophilic, even into adulthood with marked airway remodeling, increased reticular basement membrane thickness and airway smooth muscle mass (23, 57). Airway remodeling in adult-onset asthma is less well-characterized but prevalence appears to be lower than childhood-onset asthma (58). When unbiased clustering of CT airway wall thickness or airway lumen thickness was applied to an adult asthma cohort, all asthma patients manifested air trapping but one cluster with less air trapping and lacking changes consistent with airway remodeling correlated with clinically mild disease (59). Two other cohorts of adult onset asthmatics with severe disease were found to contain a subpopulation (25–30%) lacking both eosinophilic inflammation and airway remodeling (24, 50). Unbiased clustering analysis has also revealed an adult severe asthma population, identified as “paucigranulocytic” asthma, with airway remodeling in the absence of airway inflammation (28, 29, 60). In paucigranulocytic asthma, airway remodeling is thought to occur in a manner “uncoupled” from airway inflammation, perhaps as a direct consequence of airway smooth muscle hypertrophy or neurogenic factors contributing to bronchospasm (61). In most cases, however, airway remodeling seems to behave as a function of either disease severity and/or chronicity of inflammation.

### Extracellular Matrix in Asthmatic Airway Remodeling

Increased deposition of extracellular matrix (ECM) proteins in the reticular basement membrane region, lamina propria, and submucosa is a characteristic of asthmatic airways and contributes to the airway wall thickening and airflow obstruction. The ECM is composed of a diverse group of proteins and glycoproteins, including (a) structural proteins, including collagen and elastin, (b) adhesion proteins, including fibronectin and tenascin, etc, and (c) glycosaminoglycans (GAGs) and proteoglycans (62). Collagen fibers are the most abundant elements of the ECM in the lung. Fibrillar collagens, including type I, II, III, V, and XI collagens, have great tensile strength but low elasticity and contribute to the overarching architecture of the lung. Overproduction and deposition of collagen leads to lung stiffness. Elastic fibers, on the other hand, have high elasticity and provide the lung with compliance and elastic recoil. ECM adhesion proteins, such as fibronectin, provide binding sites for cell adhesion receptors including integrins. Therefore, the ECM proteins provide structural and mechanical support for lung tissue and a substratum for cell adhesion, migration, activation, and proliferation. Aberrant accumulation of ECM may, however, lead to changes in tissue structure and function that contribute to airway remodeling.

It has been well-documented that the deposition of various ECM molecules is increased in asthmatic airways, including structural proteins collagens I, III, and V, adhesion proteins fibronectin and tenascin, as well as proteoglycans such as lumican and biglycan (63–67). Fibroblasts are the major producer of ECM. Fibroblasts in asthmatic airways are activated and produce large amounts of ECM (66, 68, 69). It has also been shown that asthmatic airway epithelial cells stimulate naïve lung fibroblasts to produce collagens, fibronectin, and the pro-fibrotic mediator, TGF-β (68). Airway smooth muscle (ASM) hypertrophy and hyperplasia are characteristic features of asthmatic airways. Besides fibroblasts, smooth muscle cells in asthmatic airways also produce increased amounts of ECM, including collagens and fibronectin (70–72). Known environmental risk factors such as biomass fuels, cigarette smoke, and rhinovirus have been shown to stimulate the productions of ECM proteins by airway epithelial cells, ASM and fibroblasts (73–77). Additionally, it has been reported that the degradation products of matrix proteins, sometimes referred to as “matrikines,” regulates the remodeling process; for example, tumstatin, a type IV collagen-derived matrikine, modulates ASM production of ECM proteins (71). These matrikines, which are increased in asthmatic airways, interact with ECM proteins to regulate the composition of the matrix and modulate airway hyper-responsiveness (78, 79). Together, emerging data indicate that the deposition of ECM proteins in asthmatic airways is increased and may be post-translationally modified, which lead to specific endotypes of airway remodeling in asthma.

### Aging and Airway Remodeling

Asthma mortality has declined in the United States, but not in elderly patients. The probability of death from asthma is more than five times higher in elderly asthmatics (80–82). Aging affects the lung and chest wall, reducing FEV1 (forced expiratory volume), FEV1/FVC (forced vital capacity), and FVC (with minimal change in lung volume), and increasing residual volume (83). Age-dependent decrements in FEV1 proceed linearly from 25 to 30 years of age through adulthood, then accelerate with increasing age (84). This deterioration is further accelerated in asthmatic patients (85). Lung parenchyma structural changes affecting elastic recoil are postulated to underlie peripheral airway narrowing with reduced airway surface-to-volume ratio observed in the elderly (3, 86). Elderly patients with no known underlying lung disease also manifest alveolar dilation and ductal ectasia without emphysema or fibrosis (87). The chest wall compliance of elderly patients is reduced by costochondral joint calcification, degenerative joint disease of the spine, and kyphosis (83). Diaphragmatic weakness and skeletal muscle weakness reduce maximum inspiratory and expiratory pressures (83, 88). Comorbidities, frailty, and poor nutrition result in respiratory muscle weakness (89, 90). However, airway remodeling does not appear to occur as an intrinsic feature of aging. Rather, it is an intrinsic feature of asthma, manifesting in a subset of adult asthmatic patients.

## Airway Epithelial Cells as “Initiators” of Airway Remodeling

**Figure 1:**
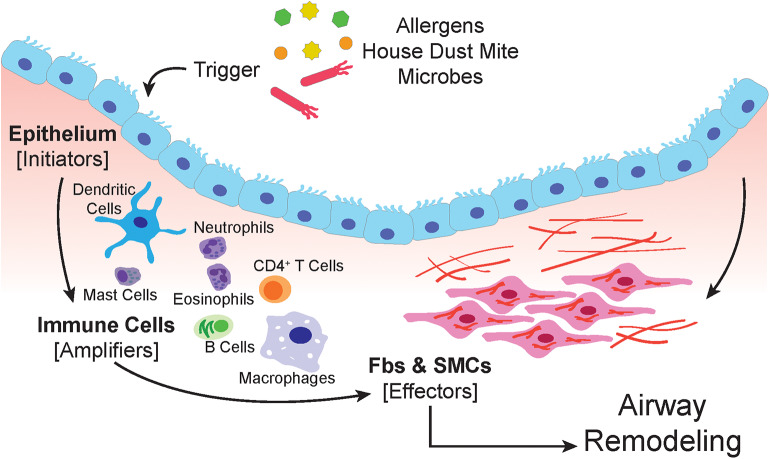
The airway epithelium serves as the primary interface between the environment and the lung. When triggered by allergens, house dust mite or microbes, epithelial cells respond by secreting soluble factors that recruit, and activate immune cells. The amplification of the immune response involves macrophages, dendritic cells, neutrophils, mast cells, eosinophils, and lymphocytes. Both the epithelium and immune cells produce paracrine signals that induce proliferation, expansion, and activation of the submucosal mesenchyme that include resident airway smooth muscle cells and fibroblasts.

The airway epithelium is subject to airborne particles and infectious agents and represents the frontline barrier between the host and environment in the airways (91) (schematic in Figure 1). Epithelial cells are armed with pattern recognition receptors (PRR) which detect pathogen-associated molecular patterns (PAMPs) and damage-associated molecular patterns (DAMPs) that are derived from pathogens, allergens, and injured cells due to environmental insults (92–95). Triggering of PRRs by allergens, results in the recruitment of dendritic cells (DCs) through the secretion of chemokines and cytokines, such as CCL2, CCL20, IL-12, IL-12p40, TSLP, and GMCSF (94, 96, 97).

The environmental insult on airway epithelial cells may also induce apoptosis, or programmed cell death (98). Apoptosis of the epithelium, accompanied by soluble paracrine factors such as TGF-β can initiate the tissue regenerative process in an attempt to restore homeostasis (99, 100). However, persistent damage and prolonged stimulation by growth factors, can lead to aberrant tissue repair and remodeling of the airways that leads to the pathophysiological conditions seen in asthma. For example, thickening of the airway smooth muscle is a result of mesenchymal differentiation into myofibroblast (101).

As the lung expands with air, the accompanying morphological changes also affects the cellular constituents of the lung (102–104). The mechanical forces experienced by cells during regular respiration is minimal; however, during bronchoconstriction and bronchospasms, epithelial cells are subject to compressive forces at a magnitude higher than normal physiological conditions (103, 105). These compressive forces may often result in mechanostimulation, which can activate epithelial cells to produce TGF-β and GMCSF, which can then recruit DCs and other immune cells (106). Recruited DCs orchestrate the activation of both innate and adaptive arms of the immune system, facilitating the inflammatory process in the airways of asthmatics (107). Mechanostimulation also increases gene expression of early growth response 1 (Egr-1), endothelin 1, transforming growth factor β1 (TGF-β1), and epidermal growth factors (EGF) (108). Secreted EGF can bind epithelial epidermal growth factor receptors (EGFR), which in turn mediates a positive feedback loop to increase EGFR ligand production (109). EGF promotes goblet-cell metaplasia (110), which also contribute to the physiological changes observed in patients exposed to repeated bronchoconstriction by methacholine challenge (25). Repeated bronchoconstriction induces goblet cell proliferation, sub-epithelial thickening, and mucus secretion which can lead to airway obstruction (25). Compressive stress also increases YKL-40 expression, encoded by the gene *CHI3L1*, and its secretion (111); this stimulates angiogenesis, smooth muscle cell proliferation, and migration (112).

Cytokines involved in asthma, such as IL-6, IL-8, and TSLP are known drivers of cellular senescence (113, 114). Cellular senescence, or the irreversible arrest of the cell cycle, may also contribute to airway remodeling and its detrimental effects on the airways of asthmatics (115). Senescent epithelial cells induced by cigarette smoke have been shown to destroy the alveoli resulting in progression of disease (116). In normal physiological setting, cellular senescence is usually a protective mechanism to prevent cells that have undergone telomere erosion and stress from proliferation and transformation (117). However, accumulation of senescent fibroblasts can contribute to reduced pulmonary compliance and remodeling of the airways (118, 119).

## Immune Cells as “Amplifiers” of Airway Remodeling

The lung, like many other organs and tissues, contain a diverse population of immune cells that protect us from pathogens invading the airways (120–122). Immune surveillance and phagocytosis facilitates the resolution of the inflammatory process back to homeostasis. However, aberrant activation and prolonged immune responses are key drivers in asthma and have detrimental effects to the airways. Notably, T helper type 2 (Th2) and Th17 cells produce cytokines that promote airway inflammation and remodeling in allergic asthma (123, 124). Th2 cytokines, such as IL-4 and IL-13 enhance subepithelial fibrosis, mucous hyperplasia, and collagen deposition (125–127). Despite the controversial role of Th17 cells in airway remodeling and inflammation, the synergistic effect of DCs together with Th17 cytokines promote accumulation of fibrotic matrix components that correlate with TGF-β expression (123).

Alveolar macrophages (AM) are important lung-resident immune cells involved in immune response and tissue repair in the lung (128). They have a protective role in maintaining pulmonary tissue homeostasis as well as phagocytosis and host defense like many other macrophages. Generally, AM dampen the inflammatory responses in the airways through phagocytosis of apoptotic bodies and clearance of innate immune cell infiltrates. In asthmatics, however, these mechanisms are impaired; thus amplified and prolonged inflammation is present in the airways. Alveolar macrophages also contribute to airway remodeling through activation by TGF-β and release of matrix metalloproteinases that alter the extracellular matrix (ECM) and airway structure.

TGF-β is strongly implicated in airway remodeling and is released by eosinophils at the site of allergic inflammation (129, 130). TGF-β promotes metalloproteinase-9 (MMP-9) production, also known as gelatinase B; a metalloproteinase found in BAL fluid as well as plasma from asthmatics (131). MMP-9 is activated by tryptase secreted from mast cells, which have been tied to hypersensitivity and allergic inflammatory responses (132). Tryptase also induces fibroblast, endothelial, and epithelial cell proliferation further fueling remodeling of the airways in asthmatics (133, 134). Neutrophils have also been shown to produce MMP-9 and are associated with severe forms of asthma (135–137). In particular, neutrophils are associated with non-allergic and steroid resistant asthma (138, 139). In non-allergic asthma, epithelial cells initiate the inflammatory process through the release of IL-6, TGF-β, and IL1-β, which stimulates the production of IL-17 (140, 141). Th17 cytokines such as IL-17 and IL-22 facilitate neutrophilic recruitment, and TGF-β production, further amplifying the inflammatory and airway remodeling responses (142–144).

In addition to cellular mediators discussed above, the complement cascade can drive the same inflammatory and remodeling responses seen in asthma. Both the classical and alternative complement pathways promote airway inflammation through recruitment of proinflammatory immune cells, such as Th2 cells, mast cells, eosinophils, and macrophages. The recruitment of these immune cells helps amplify the magnitude of airway remodeling through the release of TGF-β, IL-13, and PDGF by both immune and epithelial cells. Specifically, C3a and C5a complement molecules have been reported to stimulate pro-airway remodeling factors such as TGF-β by epithelial cells (145). These pro-remodeling factors help drive fibroblast-to-myofibroblast differentiation and the production of metalloproteinases that drive structural changes in the airways of asthmatics (145).

## Mesenchymal Cells as “Effectors” of Airway Remodeling

It is now well-recognized that resident airway smooth muscle (ASM) cells and fibroblasts drive key cellular and structural features of asthmatic airway remodeling, specifically the increase in ASM mass and subepithelial fibrosis (146). Paracrine signals from epithelial cells and immune cells may sustain mesenchymal cell activation in the airway wall (147). Bidirectional crosstalk between the epithelium and the mesenchyme is critical for normal lung development including branching morphogenesis; reactivation of this epithelial-mesenchymal tropic unit (EMTU) has been proposed as a driving mechanism in the repair response to chronic injury (148). Cytokines such as transforming growth factor-β (TGF-β) and fibroblast growth factors (FGFs) secreted by the mesenchyme instruct the growth and differentiation of epithelial cells, while epithelial growth factor (EGF), TGF-β, sonic hedgehog (SHH), and Wnt proteins from the epithelium direct the proliferation, differentiation, and fate of mesenchymal cells. An aberrantly activated EMTU in combination with inflammatory stimuli, such as the Th2 cytokines IL-4 and IL-13, may sustain the sub-mucosal mesenchymal response by ASM and fibroblasts to execute pathological airway remodeling.

Increased ASM mass has been recognized as a hallmark of airway remodeling in asthma (149, 150). There is abundant evidence for ASM plasticity, and the regulation of its proliferative, synthetic, and contractile properties (151). In support of the concept of an EMTU that recapitulates lung development, ADAM33, a membrane-anchored metalloprotease that is developmentally regulated, was identified as an asthma susceptibility gene by positional cloning in an outbred population (152). Several ADAM33 protein isoforms are expressed in human embryonic bronchi and surrounding mesenchyme, and its “reactivation” in adult ASM may explain its genetic association with asthma and bronchial hyper-responsiveness (153). In addition to pro-inflammatory/pro-fibrotic cytokines and contractile agonists that regulate ASM mass, cell intrinsic properties are also important. For example, the mitochondrial Bcl-2 adenovirus E1B 19 kDa-interacting protein, Bnip3, regulates the expression of adhesion proteins that control ASM adhesion, migration, and proliferation (154). ASM responses to β2 agonists is decreased by TGF-β1 signaling via the modulation of intracellular cAMP levels and a Smad2/3-dependent mechanism (155, 156). The TGF-β-induced activation of the reactive oxygen species (ROS)-generating enzyme, NADPH oxidase 4 (Nox4) that induces myofibroblast differentiation (157), is implicated in ASM proliferation and hypercontractility in asthma (158, 159), as well as in epithelial ciliary dysfunction in neutrophilic asthma.

Another distinct hallmark of airway remodeling in asthma is subepithelial fibrosis that is primarily mediated by submucosal resident fibroblasts that proliferate and differentiate into myofibroblasts. In addition to airway resident fibroblasts, the number, activation, and differentiation of circulating bone marrow-derived fibrocytes have been correlated with asthma severity (160). Consistently, TGF-β is recognized as a key mediator of this response, a number of paracrine mediators secreted by epithelial cells and immune cells are capable of activating submucosal fibroblasts (101). The SHH pathway has been implicated in induction of epithelial-mesenchymal transition (EMT) induced in bronchial epithelial cells by house dust mite exposure (161). The ECM itself and related proteases may serve to sustain these fibrogenic activities. For example, eosinophil-mediated fibroblast-to-myofibroblast transition and increased migration of fibroblasts is dependent on expression of matrix metalloproteinase-2 (162). In addition to pro-fibrotic cytokines such as TGF-β and Wnt proteins secreted by the epithelium, there is also evidence that epithelial-derived factors may mitigate fibrogenic responses in subepithelial fibroblasts. Club cell secretory protein-16 (CCSP-16), a member of the secretoglobin family, is decreased in serum of severe asthmatics and animal studies support a protective role of this protein against airway fibrosis and airway remodeling (162). Extracellular vesicle (EV)-mediated transfer of inositol polyphosphate 4-phosphatase type I A (INPP4A), a lipid phosphatase and an asthma candidate gene, functions to restrain the proliferative capacity of fibroblasts by dampening PI3K/Akt signaling (163). In contrast, fibroblast-derived EVs that carry fibronectin on its surface promotes invasion in recipient fibroblasts (164). Unique lipid signatures of EVs have been identified in the airways of human asthmatic subjects (165); in this study, lipidomics analysis revealed that phosphatidylglycerol, ceramide-phosphates, and ceramides were significantly reduced in exosomes from asthmatics exposed to tobacco smoke, while sphingomyelin 34:1 was more abundant in this group compared to healthy controls.

In addition to the traditional concept of targeting inflammatory responses in asthma, recent studies support potential utility in targeting the mesenchymal remodeling component. Although corticosteroids can mitigate chronic inflammation which secondarily contribute to airway remodeling, there is growing interest in developing therapies that more directly target airway fibrosis. None of the currently approved biologics, with the potential exception of IL-4/IL-13 targeted therapies, directly target cellular components of airway remodeling. However, bronchial thermoplasty is a non-pharmacological approach that may target the ASM component of airway remodeling. Bronchial thermoplasty involves the application of radiofrequency energy to the airway wall during bronchoscopy, and is thought to selectively ablate ASM; there is evidence that this procedure reduces asthma exacerbations and improves quality of life in patients with severe uncontrolled asthma (166). However, many questions remain as to its utility in severe asthma, specifically as it relates to mechanism(s) of action, patient selection, and predictors of response (167). There is likely to be advances in development of anti-fibrotic therapies for asthmatic airway remodeling. Although targeting Th2 cytokines are not particularly novel, there is continued interest in targeting this pathway in selected asthma endotypes. Recent studies suggest that epithelial cell responses to IL-4/IL-13 increases the IL-4Rα-dependent smooth muscle contribution to airway hyper-responsiveness, supporting IL-4Rα-targeted therapy in asthma (168). Activation of estrogen receptor-β signaling has been shown to downregulate airway hyper-responsiveness and airway remodeling (169). Agonists of the bitter taste receptors (TAS2Rs) promote bronchodilation, restrict allergen-induced inflammatory responses, and ASM proliferation and mitigate features of airway reactivity *in vitro* and in animal models (169, 170). Increased sphingosine kinase 2 (SPHK2) levels in proliferating ASM cells may be exploited to alleviate airway smooth muscle thickening with synthetic substrates (171, 172). When bronchodilatory responses to β-receptor agonists are blunted, the synthetic peroxisome proliferator activated receptor (PPAR)-γ agonist, rosiglitazone, may have benefit in eliciting ASM relaxation in *ex vivo* mouse lung slice models (173). Antagonism of prostaglandin D_2_ type 2 with fevipiprant reduced ASM mass in patients with asthma by decreasing airway eosinophilia in concert with reduced recruitment of myofibroblasts (174). Targeting another TGF-β-inducible gene, plasminogen activator inhibitor 1 (PAI-1), with a small molecule inhibitor has been shown to suppress eosinophilic allergic responses and ameliorate airway remodeling in an ovalbumin-sensitized murine model of chronic asthma (175).

## Aging and Inflammaging

The repercussions and effects of the aging immune system are wide-ranging and diverse. Aging, as a whole, produces complex and ubiquitous physiological alterations across nearly all organ and tissue systems leading to many age-related diseases (176–181). The human immune system relies on an intricate interplay between the innate arm of the immune system, comprised of primary sentinel immune cell populations, and adaptive responses that rely on immunologic memory (176–181). A major consequence of immune senescence is an impaired capacity to repopulate naive B cells from the bone marrow and T cells from the thymus, which involutes over time. In addition to the loss of such cellularity, there is progressive loss of functional competency of both innate and adaptive immune cells (176–182). The biological and physiological nature of immune senescence remains largely unexplored, and there is growing interest in defining mechanisms and developing novel therapeutic approaches.

Human lungs are an intricate fractal network of airways composed of the trachea, bronchioles, alveolar ducts, and terminal alveolar regions. During aging, the lung undergoes a remarkable transformation with structural and functional alterations (183–185). Concurrent with aging, a loss of bone density (osteoporosis) and muscle atrophy produce physical alterations to the spine, chest wall, and thoracic cavity. The resultant physiological and functional changes include the reduction in lung elasticity of lung tissue, forced expiratory volume (FEV), forced vital capacity (FVC), and tidal volume (TV) (183–185). Collectively, the cellular, structural, and functional changes in the aging lung results in impaired host responses to respiratory infections, higher rates of autoimmunity, and diminished capacity to repair and regenerate (183–185).

### Inflammaging

Described in 2000 by Franceschi et al., “inflammaging” is the process by which immune senescence is accompanied by low-level, chronic inflammation (186). Immune senescence is characterized by a decreased proliferative ability of cells and secretion of pro-inflammatory cytokines/mediators that is referred to as senescence-associated secretory phenotype (SASP). Replicative senescence results from a shortening of telomeres, which ultimately triggers DNA damage responses. In addition to replicative senescence, oxidative stress, epigenetic alterations, oncogene activation, and other stressors that induce DNA damage can induce cellular senescence (187). SASP activation leads to the production of pro-inflammatory cytokines such as IL-12, IL-6, IL-1β, TNFα, IFNγ, and other factors such as C-reactive protein and prostaglandins (187). The pervasive inflammatory milieu can be local or systemic in nature and is proposed to result from an accumulation of self-antigens produced by age-associated damage to tissues (186). The resultant accumulation of endogenous cellular matter induces an inflammatory environment leading to tissue destruction and injury (186). Inflammaging is well-recognized as an integral contributor to age-related disease pathology. This important recognition has led to growing interest in understanding the mechanisms that govern the inflammaging process and the subsequent effects on lung pathophysiology and age-related lung disorders. Elderly adults are more susceptible to pulmonary disorders such as asthma, Idiopathic Pulmonary Fibrosis (IPF), and COPD, however, the age-related mechanisms that drive disease pathology remains largely unresolved (90). Asthma is a disease characterized by airway inflammation, elevated mucus production, and airway obstruction (90). The disease phenotype of early-onset asthma has been well-defined and is consistent with a pattern of allergic inflammation including eosinophilia, and a Th2 bias (188). In contrast, the pathophysiology of late-onset asthma which affects the elderly is less well-understood, although inflammaging may represent one mechanism for disease susceptibility, progression, and relative obstinate responsiveness to therapy.

### Cells Implicated in Inflammaging

Macrophages resident within the alveolar compartment are a first-line defense in innate immune responses, and are implicated in inflammaging (189). Macrophages elicit varied responses depending on microenvironmental cues, and can initiate inflammation (classically activated/M1) or attenuate inflammation and promote wound healing (alternatively activated/M2) (190). In contrast to this concept of polarized macrophage phenotypes, it is now appreciated that varied phenotypes can emerge along this differentiation spectrum. Macrophages play a central role in lung homeostasis by clearing surfactant and cellular debris from apoptotic cells (191–194). During inflammaging, macrophages lose plasticity and are unable to alternate between the pro- and anti-inflammatory phenotypes (189). The age-induced alterations in macrophage function include decreased production of pro-inflammatory cytokines (IL-6, IL-1β, TNFα), deficiency in phagocytosis and cellular debris clearance, and decreased Toll-like receptor (TLR) expression in mice (195–198). This functional transformation may, in part, be responsible for creating a pervasive cycle that sustains inflammaging in the aging lung.

Naïve T helper cells, after activation, differentiate into a multitude of specialized effector subtypes of which CD4^+^ Th2 and Th17 T cells have been associated with asthma. Th2 cells and type 2 immune responses are predominately associated with early-onset asthma, although other endotypes are observed in children, highlighting the heterogeneous nature of the disease (188). The Th17 subtype is generated from naïve CD4^+^ T cells in response to IL-1β, IL-6, TGFβ, and IL-23 (199–202). They are characterized by the fate-determining transcription factor RORγt and produce IL-17a, IL-17F, and IL-22 (203, 204). Th17 cells are associated with mucosal barriers and involved in pathogen clearance (205) A new non-canonical role for Th17 cells is emerging in infection-induced asthma. IL-17 produced by Th17 cells recruit and activate neutrophils via crosstalk with airway epithelial cells (206). Additionally, it has been shown that IL-17a, IL-17f, and IL-23 promote increased mucous production, airway remodeling, and inflammation (207). Molet et al. (208) reported an increase in IL-17 in the sputum and bronchoalveolar lavage of asthmatics; they showed that IL-17 activation of macrophages and fibroblasts promoted the secretion of IL-6, IL-1β, and TNFα *in vitro*. As highlighted previously, a curious paradox exists with respect to inflammaging, an increase in immune cell senescence concurrent with chronic inflammation.

We have focused, thus far, on immune cell senescence as a regulator of immune effector cell function, chronic inflammation, and airway remodeling. An alternative view suggests that immune senescence is propagated by activation of regulatory cells possessing immunosuppressive properties. A plethora of myeloid and lymphoid-derived regulatory cells with such properties has described regulatory T cells (Tregs), regulatory B cells (Bregs), and myeloid-derived suppressor cells (MDSCs) (209–214). Regulatory T cells, defined by expression of the fate-determining transcription factor Forkhead Box P3 (Foxp3), are paramount to the maintenance of immune homeostasis (209, 210). After infectious challenge and its resolution, Tregs promote tissue repair and restrain immune hyper-reactivity through the secretion of anti-inflammatory cytokines, IL-10, and TGF-β (209–212). Tregs also express high levels of CD25 (IL-2Ra) and are thought to compete with immune effector cells for IL-2 in local inflammatory environments (205). A number of modalities of Treg-induced immunosuppression have been proposed (215). Similar to other immune cell types, Tregs precipitously decline in aged adults, the mechanism of which is incompletely understood; the role of cellular senescence in this process is likely. Traditional CD4^+^ Foxp3^+^ regulatory T cells develop in the thymus and are designated natural Tregs (nTregs) (209–212). Additionally, Tregs can be induced in the periphery from conventional CD4^+^ T cells (iTregs) (205). The frequency and total numbers of CD4^+^ Tregs are elevated during the aging process in humans (205). However, murine studies have revealed that the proportion of nTregs to iTregs increases during aging, suggesting a defect in the inducibility of Tregs from the conventional T-cell pool (205). These seemingly paradoxical observations underscore the complex nature of immune network remodeling and phenotypic switching that occurs with age.

As discussed earlier, fibroblasts represent a specialized mesenchymal cell population that produce collagen, fibronectin, and proteoglycans which comprise major components of the ECM and are found in the stroma of virtually all tissue types (216). In response to injury, fibroblasts deposit ECM components providing the physical architecture and matrix-generated signals that promote wound healing (217, 218). In age-induced pathologies such as asthma, COPD, and IPF, a reorganization of airway architecture has been consistently observed. Fibroblasts produce a number of cytokines and respond to an assortment of cytokines from neighboring cells, primarily epithelial cells and immune cells. Cross-talk between fibroblasts and immune cells has been shown to be central to disease pathologies (219). The low-level, chronic inflammation that characterizes inflammaging may alter the cytokine microenvironment in aging lung tissues; the resulting dysregulation in stromal cell-immune cell cross-talk may contribute to disease progression. The precise mechanisms driving inflammaging and its link to fibrosis and airway remodeling requires further investigation. Given the dynamism involved in the progression and pathophysiology of inflammaging, it is not surprising that the mechanistic underpinnings have yet to be fully defined. In the ensuing section, we will highlight several emerging and proposed mechanisms. Cellular senescence of ASM induced by hyperoxia leads to secretion of pro-inflammatory and pro-fibrotic mediators factors that has been proposed to contribute to pediatric airway disease in the context of sequelae of preterm birth (220, 221).

### The Role of Oxidative Stress in Inflammaging

Oxidative stress participates in myriad disease pathologies, and is particularly relevant for diseases of aging (222, 223). This biological phenomenon results from an imbalance between the production and clearance of ROS (222). ROS are produced during normal cellular processes such as oxidative metabolism, responses to bacterial infections, and during signaling events by Nox enzymes (222). ROS are important signaling mediators and during redox homeostasis participate in many cellular and physiologic processes such as proliferation, differentiation, migration, and apoptosis (222, 224). Conversely, during redox imbalance, ROS are implicated in disease pathogenesis due to excess cellular atrophy and death, macromolecule damage, and exacerbated inflammation (222) A specific source of ROS, mitochondrial ROS (mtROS), has gained considerable attention recently given the ubiquity of cellular respiration, even during homeostatic conditions. In healthy individuals, the oxidant/antioxidant balance is maintained. The oxidative stress theory of aging, proposed by Denham Harman in 1950 (225), was the first to implicate ROS in age-induced molecular alterations. This theory posits that an accumulation of reactive species (ROS/RNS) during normal cellular metabolism promotes the aging process by disturbing the redox balance in favor of pro-oxidants (225). Metabolic dysfunction and damage induces inflammaging through an innate immune sensing mechanism (226). DAMPs released following necrotic and apoptotic cell death are recognized by innate immune cells bearing PRRs such as TLRs and NOD-like receptors (NLRs) (226). Necroptosis, a form of regulated cell death, is regulated via a multiprotein signaling complex called the necrosome consisting of receptor-interacting kinase 1 (RIPK1), receptor-interacting kinase 3 (RIPK3), and mixed lineage kinase domain-like pseudokinase (MLKL) (227). This regulated cell death is also called inflammatory cell death, as necroptosis induced cell membrane rupture releases endogenous DAMPs [mitochondrial DNA (mtDNA), high-mobility group box 1 (HMGB1), genomic DNA and RNA] to the surrounding tissue microenvironment, leading to “sterile” inflammation (228). Pinti et al. (229) noted an increase in circulating mtDNA in aged adults correlated with elevated pro-inflammatory cytokines. These observations, along with previous studies, collectively suggests that maintenance of inflammaging in the aging lung may be mediated by age-induced tissue damage, release of endogenous DAMPs, and senescence of immune and non-immune cells.

### Inflammaging and the Epigenome

In addition to genetic factors, environmental and other factors may contribute to inflammaging by epigenetic mechanisms. Several epigenetic mechanisms, including direct DNA methylation, non-coding RNAs, and histone modifications, that regulate chromatin remodeling may participate in this process (230). The initiation of epigenetic modulation is triggered by diverse environment stimuli including diet, infection exposure, toxins, and the microbiome (222). Chemical modifications to histone tails leads to three-dimensional chromatin remodeling. Euchromatin, a loose, uncoiled chromatin structure, is transcriptionally permissive, while heterochromatin with a tightly packed 3-D structure is transcriptionally silent (222) While not fully understood, it is well-accepted that chemical modifications of histones such as methylation, acetylation, phosphorylation, and ubiquitination alter chromatin structure. Methylation and acetylation of histone tails, the most studied of the modifications, are associated with permissive (H3K4me3, H3K9me1, H3K9ac, and H3K27ac) in addition to, repressive (H3K9me3 and H3K9me3) histone signatures (222). Ultimately, these chemical modifications influence gene expression in a dynamic interplay that also requires specific transcription factors and co-factors. Age-dependent and ubiquitous hypomethylation of DNA and heterochromatin loss have been broadly reported (222, 231). Furthermore, age-related alterations in post-translational modifications of histone tails have been detailed and a ubiquitous loss of nucleosome density has been observed (231). The direct relationship between age-driven epigenetic remodeling and inflammaging in pulmonary tissues is incompletely understood. It has been proposed that, in aging human cells, loss of heterochromatin is due to diminished nucleosome occupancy (231). In fact, (232), first observed this phenomenon in human skin fibroblasts (231). It was reported by Agrawal et al. (233) that DNA from elderly adults is more immunogenic relative to aged controls. Moreover, it was speculated that DNA from aged adults is a more potent DAMP due to hypomethylation, thus indistinguishable from microbial DNA by PRRs expressed by innate immune cells (222).

### Metabolism and Inflammaging

Metabolic dysfunction is implicated in many age-associated chronic diseases and has been suggested to not only be a result of inflammaging, but also directly contributes to the aging process (234). Both innate and adaptive immune responses have been demonstrated to be regulated via the metabolism of multiple amino acids including tryptophan, arginine, phenylalanine, cysteine, and glutamine (235). Arginine has been shown to play a critical role in the pathogenesis of allergen-driven asthma (236–238). The catabolism of arginine via arginase is not only a biomarker for the onset of asthma, but it has been suggested to be directly involved in the manifestation of allergic airway disorders (236–238). Arginine is the substrate for both nitric oxide synthase (NOS) and arginase. These enyzmes regulate each other by controlling the availability of the substrate arginine (236–238). Given the opposing regulatory functions of NOS and arginase, a better understanding of this dynamic equilibrium in allergy-driven asthma is required (239, 240). In a 2015 study, Comhair et al. (241) identified 25 metabolic intermediates that were significantly different in the plasma of asthmatics vs healthy controls. Furthermore, their work revealed that severe asthmatics demonstrated lower levels of steroid metabolism intermediates. Alternatively, increased plasma levels of taurine, bile acids, nicotinamide, arachidonate, and adenosine-5-phosphate were observed (241). Collectively, these studies and others, underscore how the alterations in tightly regulated metabolic and homeostatic processes in aging individuals may increase susceptibility to allergic asthma.

The role of lipid metabolism in age-related diseases is not well-understood. However, it has been reported that plasma triglyceride levels increase, while phosphatidylethanolamine (PE) and phosphatidylcholine (PC), which are generally associated with membranes, decrease with age (242). Moreover, Lawton et al. (243) reported the age-related modulation of lipid composition including elevated levels of fatty acids, beta-hydroxybutyrate, carnitine, and cholesterol in older individuals. The relationship between disturbed lipid metabolism dynamics and inflammatory responses remain poorly understood. Three isoforms of the nuclear receptor family of transcription factors- peroxisome proliferation-activated receptors exist: PPARa, PPARb, and PPARg (244, 245). Fatty acid metabolism is facilitated ultimately through alterations in transcription of PPAR sensitive genes, thus PPARs function as fatty acid sensors (244, 245). PPARg has been shown to down-modulate gene expression in both monocytes and macrophages, inhibiting their activation and production of pro-inflammatory cytokines (246–248). Lipid metabolism can also regulate chromatin remodeling by direct modification of histone tails (242). Fatty acid beta-oxidation produces multiple intermediates including acetyl CoA (242). Acetyl CoA is a required cofactor used by histone acetyltransferases in order to acetylate histone tails (242). S-adenosyl methionine (SAM) is a common co-substrate and acts primarily as a methyl donor for many physiological processes such as histone and DNA methylation, as well as lipid methylation (249). The role of fatty acid metabolism in inflammaging is likely complex and may involve coordinated regulation of the epigenome with diverse biological processes altered during aging.

Loss of homeostasis in aging tissues, including the lung, is characterized by progressive metabolic dysfunction and physiological decline in aged populations (250). Aging-related alterations in metabolic function include mitochondrial dysfunction, hyperlipidemia, and increased production of ROS, all of which are implicated in inflammatory processes as well. The relationship between aging pulmonary tissues, metabolic function, and inflammation is nuanced and complex, underscoring the need for additional research into the mechanistic underpinnings of inflammaging in the lung.

## Conclusion

**Figure 2:**
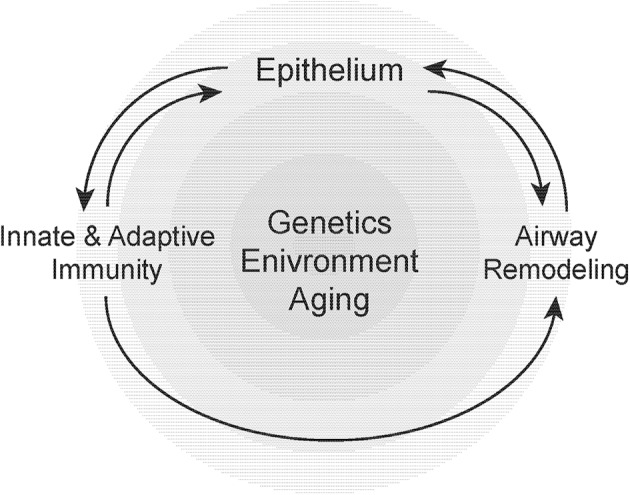
Susceptibility to airway remodeling in individual hosts is dependent on genetic susceptibility, environmental exposures, and aging. These risk factors regulate the cross-talk between the epithelium, innate, and adaptive immunity and mesenchymal stromal cells that contribute to airway remodeling.

The development of asthma, its progression and associated physiological declines in lung function is dependent on a number of genetic, environmental, and host-related factors that include age (251, 252) (Figure 2). Our understanding of the epidemiology, clinical behavior/prognosis, and the cellular/molecular pathogenesis of asthma have advanced over the past decade. Most notably, the recognition and improved understanding of disease subphenotypes and pathological endotypes has informed the need for greater precision in both the diagnosis and treatment of these heterogeneous group of clinical “syndromes.” Airway remodeling may be viewed as a specific endotype of asthma pathology that is relatively refractory to conventional anti-inflammatory therapies. It has been proposed that corticosteroid refractory asthma may represent a sub-phenotype characterized by a heightened neutrophilic airway inflammatory response in the presence or absence of eosinophils, with evidence of increased tissue injury and remodeling (253). Asthma in the aging population appears to share several features with this group of corticosteroid-refractory asthma. Recognizing that airway remodeling may occur in parallel with chronic inflammation, and not simply as a (serial) consequence of the inflammatory response, will be critical to developing novel therapeutic strategies. Over the past several years, we have witnessed intensified efforts by both academia and industry to more specifically target airway remodeling events in disease pathogenesis. It is our hope that such efforts will lead to the discovery and development of more effective therapies for severe, steroid-resistant asthma.

## Author Contributions

KH wrote sections and drew figures for manuscript. JD and VT formulated the outline of the review, wrote sections of the manuscript, and edited the manuscript. MC and TB wrote sections of the manuscript. JT and R-ML edited the manuscript. All authors contributed to the review.

## Conflict of Interest

The authors declare that the research was conducted in the absence of any commercial or financial relationships that could be construed as a potential conflict of interest.

## Footnotes

^1^Available online at: http://www.nhlbi.nih.gov/health-pro/guidelines/current/asthma-guidelines

^2^Available online at: www.ginathma.org
